# Technical Efficiency of Ayushman Arogya Mandirs and Their Determinants in Rural Central India: A Data Envelopment Analysis

**DOI:** 10.7759/cureus.114183

**Published:** 2026-08-08

**Authors:** Ananthakrishnan M, Chaitra C M, Deepti Dabar, Abhijit P Pakhare, Ankur Joshi

**Affiliations:** 1 Department of Community and Family Medicine, All India Institute of Medical Sciences, Bhopal, Bhopal, IND

**Keywords:** ayushman arogya mandir (aam), ayushman arogya mandirs, ayushman bharat, community health officer (cho), data envelopment analysis (dea), primary health care, technical efficiency

## Abstract

Background

Ayushman Arogya Mandirs (AAMs), formerly known as Health and Wellness Centres (HWCs), were established under the Ayushman Bharat initiative to strengthen primary healthcare delivery through Comprehensive Primary Health Care (CPHC) services in India. Assessing technical efficiency (TE) is important to understand how effectively these centres utilize available resources to deliver healthcare services and achieve desired outputs. The present study was conducted to evaluate the TE of AAMs and identify factors associated with their efficiency in a rural block of Madhya Pradesh, India.

Methods

The study included all 25 AAMs functioning in the Obedullaganj block of Madhya Pradesh during the 2022-2023 financial year. Secondary data on human resources, logistics, training, outpatient services, maternal and child health services, and non-communicable disease services were collected from health records and government health portals. An output-oriented Data Envelopment Analysis (DEA) model was used to estimate the TE of AAMs. A Tobit regression model was subsequently applied to identify institutional and individual factors associated with the TE scores.

Results

Among the 25 AAMs included in the study, six (24%) were identified as relatively efficient, while the remaining centres demonstrated varying degrees of inefficiency. The mean TE score among the low-performing AAMs was 0.66 (SD: 0.16), indicating that these centres could potentially increase their outputs by 34% using the existing level of inputs. Most AAMs had adequate staffing availability; however, deficiencies in medicine availability, training coverage, and achievement of service delivery targets were observed. Tobit regression analysis showed that the age of the Community Health Officer (CHO), total population served by the AAM, and duration since the CHO had been posted at the centre were significantly associated with the TE scores.

Conclusion

The study demonstrates the feasibility of assessing the TE of AAMs using DEA and highlights substantial variations in performance across centres. Identifying factors associated with efficiency can support targeted interventions and resource optimization strategies to strengthen primary healthcare delivery at the block and district levels.

## Introduction

India has consistently emphasized the importance of Universal Health Coverage (UHC), with a focus on ensuring equitable access to essential healthcare services without causing financial hardship to the population [1]. The National Health Policy (NHP) 2017 identified strengthening primary healthcare as a key strategy for achieving UHC in the country [2]. In line with this vision, the Government of India launched the Ayushman Bharat initiative, under which Health and Wellness Centres (HWCs), recently renamed as Ayushman Arogya Mandirs (AAMs), were established to deliver Comprehensive Primary Health Care (CPHC) services [3].

AAMs represent a major reform in India’s primary healthcare system and aim to improve the accessibility, continuity, and quality of healthcare services at the community level. These centres provide a broad range of services, including maternal and child healthcare, management of communicable and non-communicable diseases, elderly care, palliative care, and health promotion activities [4]. Each AAM is led by a Community Health Officer (CHO), supported by Multipurpose Workers (MPWs) and Accredited Social Health Activists (ASHAs), to ensure effective delivery of primary healthcare services and appropriate referral linkages [5,6].

Despite several healthcare initiatives and improvements in key health indicators, India continues to face substantial challenges in achieving universal and equitable healthcare coverage. Variations in healthcare infrastructure, workforce availability, supply chain management, and community participation contribute to disparities in service delivery across regions [7,8]. In addition, the growing burden of non-communicable diseases has increased the demand for efficient and responsive primary healthcare systems [9].

Efficient utilization of available resources is essential in resource-constrained healthcare settings such as India. Technical efficiency (TE) refers to the ability of a healthcare facility to maximize outputs using the available inputs and is an important indicator of health system performance [10]. Evaluating the TE of healthcare facilities can help identify underperforming centres, optimize resource utilization, and guide evidence-based policy decisions [11]. Data Envelopment Analysis (DEA), a widely used non-parametric analytical method, enables the assessment of relative efficiency among comparable healthcare units using multiple input and output indicators [12].

Although AAMs play a central role in delivering primary healthcare services in India, evidence regarding their operational efficiency remains limited. Hence, the primary objectives of this study were two-fold: (i) to evaluate the TE of AAMs in Obedullaganj block of Raisen district, Madhya Pradesh, using an output-oriented DEA model under Variable Returns to Scale (VRS); and (ii) to conduct a second-stage Tobit regression analysis to examine the non-discretionary environmental and operational factors associated with the derived TE scores.

## Materials and methods

Study design and settings

A cross-sectional study was conducted in the Obedullaganj block of Raisen district, Madhya Pradesh, India. The block serves as the rural field practice area of the Department of Community and Family Medicine, All India Institute of Medical Sciences (AIIMS), Bhopal, India. The study area covers a population of approximately 238,000 residing across 240 villages. The healthcare infrastructure of the block includes three Community Health Centres (CHCs), three Primary Health Centres (PHCs), and 32 Sub-centres, of which 25 had been upgraded to AAMs. The study utilized secondary data collected for the period from April 1, 2022, to March 31, 2023.

Study population and sampling

All 25 AAMs functioning in the Obedullaganj block during the study period were included as decision-making units (DMUs) for the efficiency analysis using a census-based approach. AAMs with complete and consistent data on the selected input and output indicators were included in the study.

Data collection and variable selection

A desk review of national guidelines, operational manuals, and published literature on healthcare efficiency studies was conducted to identify appropriate input and output variables for the analysis [13-14]. Secondary data were collected from reports submitted by CHOs, facility records maintained at AAMs, and government health portals, including the Ayushman Bharat Health and Wellness Centre (AB-HWC) portal, the Reproductive and Child Health (RCH) portal, the Nikshay portal, and the Comprehensive Primary Health Care-Non-Communicable Diseases (CPHC-NCD) application.

The selected input variables represented three major domains: human resources, logistics, and capacity building. These included the percentage of staff available as per sanctioned norms, percentage availability of essential medicines, and number of training sessions attended by CHOs during the previous year.

The output variables represented key service domains of AAMs, including outpatient services, maternal and child health services, and NCD services. Five different combinations of output indicators were tested to assess the robustness of the model. Spearman’s rank correlation analysis was performed to assess inter-correlation among variables before finalizing the output indicators. The final output variables included total outpatient department (OPD) consultations, antenatal care (ANC) visits provided, percentage of fully immunized children, number of NCD screenings conducted, and number of NCD patients receiving follow-up treatment.

The number of variables included in the DEA model was restricted in accordance with methodological recommendations to ensure adequate discriminatory power relative to the number of DMUs [15].

Statistical analysis

TE of the AAMs was estimated using an output-oriented DEA model. DEA is a non-parametric linear programming technique used to assess the relative efficiency of comparable DMUs by evaluating their ability to maximize outputs using available inputs [16]. An output-oriented DEA model under VRS was implemented using the benchmarking package in R. The output-oriented approach was selected because health facility managers in public sector settings have limited control over input reductions (e.g., personnel or government supply infrastructure) but can optimize service delivery outputs. VRS was specified to ensure that performance comparisons were made among facilities operating at similar scales of operation.

Following the DEA, Tobit regression analysis was performed to identify institutional and individual factors associated with TE scores. Because TE scores derived from DEA are continuous variables restricted to the interval (0,1), standard OLS regression yields biased and inconsistent estimates. Therefore, a two-tailed Tobit regression model (censored at 1) was employed to model the association between facility contextual attributes and TE [17]. The TE score of each AAM was considered the dependent variable, while explanatory variables included age and educational qualification of the CHO, duration since posting at the AAM, population covered by the AAM, and building ownership status. Statistical analysis was performed using the deaR package in R software version 4.1.3 (R Foundation for Statistical Computing, Vienna, Austria).

Ethical considerations

The study was approved by the Institutional Human Ethics Committee of AIIMS Bhopal (Approval No: IHEC-LOP/2020/PG/July/08). The study utilized secondary programmatic data, and necessary administrative permissions were obtained prior to data collection.

## Results

A total of 25 AAMs functioning in the Obedullaganj block were included in the study. Each AAM catered to a population of approximately 4,500-6,000 individuals. All 25 centres had a designated CHO, of whom 23 (92%) were female. Most CHOs were graduates in nursing (n = 21, 84%), while the remaining were AYUSH practitioners (n = 4, 16%). Nearly half of the CHOs had two or more years of work experience, whereas 10 (40%) had less than one year of experience.

The distribution of input indicators across AAMs is presented in Table 1. Complete staffing as per norms was observed in 15 AAMs (60%), while 23 centres (92%) had at least 75% staff availability. Availability of essential medicines varied considerably across centres. Only one AAM (4%) had more than 90% availability of essential medicines, whereas five centres (20%) had less than 50% medicine availability. Variations were also observed in capacity-building activities, with only eight CHOs (32%) attending at least 12 training sessions during the previous year.

**Table 1:** Distribution of AAMs According to Input Indicators Related to Human Resources, Logistics, and Capacity Building AAM: Ayushman Arogya Mandir; CHO: Community Health Officer

Main Domain	Subdomain	Number of AAMs (N = 25)
Human Resources (staff availability as per prescribed norms)	100% staff availability	15 (60%)
	75-100% staff availability	8 (32%)
	Less than 75% staff availability	2 (8%)
Logistics (availability of essential medicines)	More than 90% medicine availability	1 (4%)
	50-90% medicine availability	19 (76%)
	Less than 50% medicine availability	5 (20%)
Capacity Building (training sessions attended by CHOs during the previous year)	At least 12 training sessions	8 (32%)
	8-10 training sessions	13 (52%)
	Less than 8 training sessions	4 (16%)

The performance of AAMs across selected output indicators is summarized in Table 2. Only three AAMs (12%) achieved the annual target for OPD consultations, while seven centres (28%) achieved less than 50% of the target. With respect to maternal and child health services, only one AAM (4%) achieved full ANC coverage among registered pregnant women, and none of the centres achieved the target for full immunization coverage. However, 20 AAMs (80%) provided ANC services to at least 75% of registered pregnant women. Regarding NCD services, nine AAMs (36%) screened less than 50% of individuals aged 30 years and above for hypertension. In contrast, follow-up care for hypertension was relatively better, with 24 centres (96%) providing follow-up treatment to more than 90% of diagnosed patients.

**Table 2:** Distribution of AAMs According to Achievement of Selected Service Delivery Targets Based on AB-HWC Guidelines AAM: Ayushman Arogya Mandir; AB-HWC: Ayushman Bharat Health and Wellness Centre; OPD: Outpatient Department; ANC: Antenatal Care

Output Indicator	Target as per AB-HWC Guidelines	Achievement Level Among AAMs			
		100%	75-100%	50-75%	<50%
OPD consultations conducted	80 OPD consultations per 1,000 population	3 (12%)	7 (28%)	8 (32%)	7 (28%)
Pregnant women receiving full antenatal care (ANC)	100% of registered pregnant women	1 (4%)	19 (76%)	4 (16%)	1 (4%)
Children fully immunized	100% immunization coverage as per schedule	0 (0%)	3 (12%)	16 (64%)	6 (24%)
Hypertension screening among individuals aged ≥30 years	80% screening coverage within one year	0 (0%)	9 (36%)	7 (28%)	9 (36%)
Regular follow-up and treatment of hypertensive patients	Follow-up care for at least 50% of diagnosed hypertensive patients	11 (44%)	14 (56%)	0 (0%)	0 (0%)

The TE scores obtained through DEA are presented in Table 3. Six AAMs (24%) were identified as technically efficient, each with a TE score of 1.0. The mean TE score across all evaluated AAMs was 0.66 (SD ± 0.18). Under an output-oriented DEA framework, this indicates that inefficient centres operate at 66% of their maximum technical potential, implying an output expansion potential of approximately 51.5% without requiring additional input resources. The remaining centres demonstrated varying degrees of inefficiency. The mean TE scores of Category II, Category III, and Category IV AAMs were 0.85, 0.66, and 0.50, respectively. These findings indicate that lower-performing AAMs could potentially increase service outputs by approximately 34% using the existing level of resources.

**Table 3:** Technical Efficiency Scores of AAMs in Obedullaganj Block Based on Data Envelopment Analysis TE: Technical Efficiency; AAM: Ayushman Arogya Mandir

Efficiency Category	AAM	Technical Efficiency Score
Category I (Fully efficient centres; TE score = 1.00)	AAM 6	1.000
	AAM 8	1.000
	AAM 13	1.000
	AAM 21	1.000
	AAM 22	1.000
	AAM 25	1.000
Category II (Moderately efficient centres; TE score = 0.75-0.99)	AAM 2	0.923
	AAM 3	0.922
	AAM 5	0.885
	AAM 9	0.828
	AAM 10	0.783
	AAM 1	0.768
Category III (Partially efficient centres; TE score = 0.60-0.74)	AAM 4	0.748
	AAM 7	0.665
	AAM 11	0.660
	AAM 12	0.658
	AAM 14	0.642
	AAM 15	0.602
Category IV (Low-efficiency centres; TE score <0.60)	AAM 16	0.581
	AAM 17	0.580
	AAM 18	0.577
	AAM 19	0.520
	AAM 20	0.473
	AAM 23	0.449
	AAM 24	0.344

A robustness analysis was performed using five different combinations of output variables, and the results are presented in Table 4 and Table 6 in Appendix 1. The efficiency scores of the six high-performing AAMs remained consistently at 1.0 across all model permutations, while only minimal variations were observed among the lower-performing centres, indicating the stability and robustness of the DEA model used in the study.

**Table 4:** Combinations of Output Indicators Used for Robustness Analysis of the Data Envelopment Analysis Model OPD: Outpatient Department; ANC: Antenatal Care; HTN: Hypertension; DM: Diabetes Mellitus; TB: Tuberculosis

Variables Selected	Set 1	Set 2	Set 3	Set 4	Set 5
OPD Target Achieved	✓	X	✓	X	✓
Estimated Pregnancies Registered	X	X	✓	X	✓
Full ANC Service Given	✓	✓	X	✓	X
Fully Immunized Children	✓	✓	X	✓	X
HTN Screened	✓	X	✓	✓	X
HTN Treated	✓	✓	X	X	✓
DM Screened	X	✓	✓	X	✓
DM Treated	X	X	✓	✓	X
TB Cases Referred	X	✓	X	✓	✓

The determinants of TE identified through Tobit regression analysis are shown in Table 5. The age of the CHO and the total population served by the AAM were negatively associated with TE scores, whereas longer duration since CHO posting was positively associated with efficiency. Although not statistically significant, AAMs operating in rented buildings tended to demonstrate lower efficiency scores compared to centres functioning in government-owned buildings.

**Table 5:** Tobit Regression Analysis Showing Factors Associated With Technical Efficiency Scores of AAMs CHO: Community Health Officer; AAM: Ayushman Arogya Mandir

Independent Variable	Regression Coefficient (β)	Standard Error	p-value
Age of Community Health Officer (CHO)	-0.0239	0.0149	0.023
Duration since CHO posting at the AAM	0.0109	0.0396	0.002
Educational qualification of CHO	0.0232	0.0876	0.233
Total population covered by the AAM	-0.0393	0.0120	0.010
AAM functioning in a rented building	-0.0485	0.0727	0.504

## Discussion

The present study assessed the TE of AAMs in a rural block of central India using DEA and explored factors associated with efficiency using Tobit regression analysis. The findings demonstrated substantial variations in efficiency across AAMs, despite the centres operating within a similar administrative and healthcare setting. While a few centres achieved optimal efficiency, several AAMs showed considerable scope for improving service delivery using the existing level of resources.

The study identified notable disparities in the availability of essential resources across AAMs. Although most centres had adequate staffing levels as per prescribed norms, the availability of essential medicines was suboptimal in several centres. Medicine shortages may adversely affect continuity and quality of primary healthcare services, particularly for chronic disease management and maternal and child healthcare services. Similar findings from previous studies have highlighted the importance of strong supply chain systems and uninterrupted logistics support in improving primary healthcare performance [18].

Capacity-building activities among CHOs also varied considerably across centres. Only a limited proportion of CHOs attended the recommended number of training sessions during the previous year. Continuous training and supportive supervision are essential for strengthening the competencies of frontline healthcare providers, especially in the context of expanding primary healthcare responsibilities under the Ayushman Bharat initiative. Previous studies have reported that regular skill enhancement and mentorship contribute significantly to improved healthcare delivery and program implementation [19,20].

The analysis of output indicators revealed that many AAMs were unable to achieve key service delivery targets, particularly for outpatient consultations, immunization coverage, and hypertension screening. However, follow-up care for diagnosed hypertensive patients was relatively better across most centres. This finding suggests that while continuity of care mechanisms may be functioning adequately, gaps persist in population-level screening and outreach activities. Inadequate community mobilization, high workload, limited manpower, and logistical constraints may have contributed to the lower coverage of preventive and promotive healthcare services.

The DEA findings demonstrated that only six AAMs were technically efficient, while the remaining centres showed varying levels of inefficiency. The lower-performing AAMs had the potential to improve outputs substantially without requiring additional resources. These findings emphasize that differences in operational practices, resource utilization, and local management strategies may significantly influence performance. Similar observations have been reported in other healthcare efficiency studies using DEA, where variations in managerial efficiency and service organization contributed to differences in TE among comparable health facilities [21,22].

The Tobit regression analysis identified several factors associated with TE. Increasing age of CHOs and larger population coverage were negatively associated with efficiency scores, whereas longer duration of posting at the same centre was positively associated with efficiency. Longer tenure at a particular facility may improve familiarity with community health needs, local healthcare systems, and service delivery workflows, thereby enhancing operational efficiency. Conversely, centres serving larger populations may experience higher workloads and resource strain, which could adversely affect service delivery performance. These findings are consistent with previous research emphasizing the importance of appropriate workforce distribution and contextual resource allocation in strengthening primary healthcare systems [23,24].

Although not statistically significant, centres operating in rented buildings demonstrated relatively lower efficiency scores. Infrastructure constraints in rented facilities may affect service organization, storage of medicines and equipment, privacy during consultations, and overall patient experience. Improving infrastructure and ensuring functional facility readiness may therefore contribute to better healthcare delivery at the primary care level.

A closer examination of the six technically efficient AAMs reveals distinct operational and contextual characteristics that set them apart from underperforming facilities. Efficient centres were predominantly managed by CHOs with longer facility tenures, suggesting that staff stability fosters stronger community rapport, streamlined daily workflows, and better patient follow-up. Furthermore, these benchmark facilities maintained higher average essential medicine availability and operated mostly out of government-owned infrastructure, which facilitated organized drug storage and uninterrupted clinic operations. In terms of service outputs, efficient AAMs achieved a more balanced workload, sustaining high OPD volume alongside consistent outreach targets for NCD screenings and routine immunization without overwhelming the available health workforce. Conversely, inefficient centres frequently experienced supply-chain bottlenecks, higher CHO turnover or shorter posting durations, and severe workload imbalance driven by disproportionately large catchment populations.

The findings of the present study have important policy implications. Regular assessment of TE can help identify underperforming centres and facilitate targeted interventions for improving service delivery. Strengthening medicine supply systems, enhancing supportive supervision and training for CHOs, optimizing workload distribution, and improving healthcare infrastructure may contribute to improved efficiency and better utilization of available resources. In addition, efficient AAMs identified through DEA may serve as benchmark centres for cross-learning and dissemination of best practices within the health system.

This study has certain limitations. First, the analysis was conducted in a single rural block of Madhya Pradesh, and the sample size was low, which may limit the generalizability of the findings to other settings. Second, the study relied on secondary programmatic data, which may be subject to reporting inaccuracies and incomplete documentation. Third, DEA measures relative efficiency within the selected DMUs and is sensitive to the selection of input and output variables. Similarly, while Tobit regression accounts for the censored nature of DEA efficiency scores (0 ≤ θ ≤ 1), two-stage DEA-Tobit frameworks can be sensitive to small sample sizes and may introduce serial correlation among efficiency scores. Furthermore, qualitative dimensions of healthcare delivery, such as patient satisfaction, quality of care, and community participation, could not be assessed in the present study. Despite these limitations, the study provides valuable evidence regarding the operational efficiency of AAMs in resource-constrained settings.

Future research should focus on integrating more nuanced population demographics and socioeconomic data to better understand the disparities in AAM performance. This approach will not only improve TE but also ensure that AAMs fulfil their mandate of delivering equitable and accessible healthcare to underserved populations.

## Conclusions

The present study demonstrated considerable variation in the TE of AAMs operating within the same rural healthcare setting. While a few centres achieved optimal efficiency, several AAMs showed substantial potential to improve service delivery using the existing level of resources. Factors such as the age and duration of posting of CHOs, as well as the population served by the centres, were found to significantly influence TE.

The findings highlight the feasibility and utility of applying DEA to evaluate the performance of primary healthcare facilities at the community level. Regular efficiency assessment can support evidence-based planning, identify underperforming centres, and guide targeted interventions to optimize resource utilization. Strengthening medicine availability, capacity-building activities, supportive supervision, and infrastructure may help improve the performance of AAMs and contribute to more effective delivery of comprehensive primary healthcare services.

## Appendices

Appendix 1

**Table 6:** Distribution of Technical Efficiency Scores of AAMs Across Five Different DEA Model Configurations AAM: Ayushman Arogya Mandir; DEA: Data Envelopment Analysis

AAM	Set 1	Set 2	Set 3	Set 4	Set 5
AAM 6	1.000	1.000	1.000	1.000	1.000
AAM 8	1.000	1.000	1.000	1.000	1.000
AAM 13	1.000	1.000	1.000	1.000	1.000
AAM 21	1.000	1.000	1.000	1.000	1.000
AAM 22	1.000	1.000	1.000	1.000	1.000
AAM 25	1.000	1.000	1.000	1.000	1.000
AAM 2	0.923	0.846	0.943	0.897	0.935
AAM 3	0.922	0.880	0.889	0.953	0.906
AAM 5	0.885	0.823	0.833	0.873	0.939
AAM 9	0.828	0.877	0.768	0.795	0.812
AAM 10	0.783	0.813	0.823	0.763	0.753
AAM 1	0.768	0.822	0.832	0.772	0.836
AAM 4	0.748	0.757	0.777	0.807	0.744
AAM 7	0.665	0.668	0.679	0.718	0.673
AAM 11	0.660	0.699	0.735	0.712	0.693
AAM 12	0.658	0.699	0.709	0.649	0.735
AAM 14	0.642	0.635	0.645	0.685	0.631
AAM 15	0.602	0.635	0.645	0.585	0.611
AAM 16	0.581	0.577	0.587	0.527	0.565
AAM 17	0.580	0.587	0.568	0.537	0.545
AAM 18	0.577	0.576	0.536	0.576	0.578
AAM 19	0.520	0.523	0.546	0.553	0.523
AAM 20	0.473	0.435	0.545	0.485	0.434
AAM 23	0.449	0.425	0.435	0.475	0.440
AAM 24	0.344	0.379	0.389	0.359	0.364
